# A Split‐Face Randomized Study on the Efficacy of a Platinum‐Liposome‐Based Facial Mask Containing Soothing Ingredients for Post‐Photorejuvenation Skin Recovery

**DOI:** 10.1111/jocd.70452

**Published:** 2025-09-16

**Authors:** Zhixin Du, Lu Ren, Hui Liang, Yongjie Lu, Dongying Zhang, Zhanghao Li, Shuangyan Wang, Kangjin Zhang, Ying Wu, Dongcui Li, Li Ye, Naisheng Jiang

**Affiliations:** ^1^ Hua An Tang Biotech Group Co., Ltd. Guangzhou China; ^2^ Key Laboratory of Advanced Materials and Devices for Post‐Moore Chips, Ministry of Education, School of Materials Science and Engineering University of Science and Technology Beijing Beijing China; ^3^ Dermatology Hospital Southern Medical University Guangzhou China; ^4^ NMPA Key Laboratory for Safety Evaluation of Cosmetics Southern Medical University Guangzhou China

**Keywords:** anti‐inflammatory, facial mask, photorejuvenation, platinum liposomes, sensitive skin, skin barrier repair, skin hydration

## Abstract

**Background:**

Photorejuvenation is commonly employed to improve skin appearance but frequently leads to transient irritation and temporary impairment of skin barrier function. Incorporating platinum (Pt)‐liposome technology along with soothing ingredients such as panthenol, dipotassium glycyrrhizate, madecassoside, and *Portulaca oleracea* extract is expected to offer enhanced reparative and anti‐inflammatory effects, helping to alleviate post‐procedural skin sensitivity and restore barrier integrity.

**Aims:**

This study aimed to evaluate the efficacy of a Pt‐liposome‐based facial mask in promoting skin recovery and soothing irritation post‐photorejuvenation. Furthermore, the study assessed potential synergistic benefits from combining Pt‐liposomes with established soothing agents.

**Patients/Methods:**

We initially assessed the reparative effects of Pt‐liposomes using a 3D epidermal skin model and normal human epidermal keratinocytes (NHEK), evaluating parameters such as stratum corneum thickness, cholesterol content, ceramide chain length, and inflammatory responses (IL‐8 mRNA expression) following lipopolysaccharide (LPS) stimulation. A randomized, split‐face clinical trial involving 30 subjects who underwent photorejuvenation treatment was then conducted. Each participant applied the Pt‐liposome‐infused facial mask to one side of the face and a control product on the other side. Skin hydration, transepidermal water loss (TEWL), and erythema, tightness, dryness, and scaliness were assessed using objective instrumentation and subjective evaluations at baseline and various intervals up to 14 days post‐treatment.

**Results:**

In vitro testing showed that Pt‐liposomes significantly increased stratum corneum thickness, cholesterol levels, and ceramide chain length (*p* < 0.01). Pt‐liposomes also reduced histamine‐induced calcium influx in NHEK cells (*p* < 0.01). In LPS‐stimulated THP‐1 cells, combined treatment with Pt‐liposomes and soothing agents resulted in a greater reduction in IL‐8 mRNA expression compared to either component alone (*p* < 0.01). Clinical measurements indicated that the application of the Pt‐liposome‐based facial mask significantly increased skin hydration and reduced TEWL compared to control (*p* < 0.001) from Day 1 to Day 14. Subjective and dermatological evaluations showed statistically significant improvements in erythema, tightness, dryness, and scaliness on the treated side at all measured time points. No adverse reactions were reported.

**Conclusion:**

The Pt‐liposome‐infused facial mask can effectively promote skin barrier repair, alleviate irritation, and enhance hydration following photorejuvenation. Its synergistic combination with soothing ingredients provides rapid relief from irritation and sustained therapeutic benefits, supporting its potential as a safe and effective option for post‐procedural skincare.

## 1 Introduction

Photorejuvenation, a widely used cosmetic treatment, employs intense pulsed light (IPL) to improve skin appearance by targeting pigment and stimulating collagen production through thermal effects. While generally safe, improper control of treatment parameters can lead to side effects such as transient pain, erythema, and scaling [1]. To promote recovery and minimize irritation, a targeted skincare solution is often needed. Because post‐photorejuvenation skin is more fragile and prone to irritation, soothing care should target three primary concerns: (1) compromised skin barrier integrity, (2) cutaneous nerve hyperresponsiveness, and (3) inflammation. Several ingredients are known to provide repairing, soothing, and moisturizing benefits, including dexpanthenol, dipotassium glycyrrhizate, madecassoside, and *Portulaca oleracea* extract. Dexpanthenol is a well‐known compound that can enhance skin hydration, strengthen the skin barrier, and promote wound healing [2]. Dipotassium glycyrrhizate, derived from glycyrrhizic acid, possesses anti‐inflammatory, antioxidant, and antibacterial properties with minimal risk of allergic reaction [3]. Madecassoside, which is the principal active compound in *Centella asiatica* , can promote cell growth, accelerate wound healing, and reduce inflammation [4]. *Portulaca oleracea* extract, on the other hand, is a herbaceous plant‐derived ingredient with well‐documented anti‐inflammatory [5], antioxidant [6], and wound‐healing [7] properties.

In addition to the aforementioned soothing ingredients, platinum (Pt) particles are recognized for their skin‐beneficial properties, including antioxidant [8, 9], anti‐inflammatory [10], antibacterial [11, 12, 13], and collagen‐stimulating effects [14, 15]. Their antioxidant properties can help to neutralize free radicals, thereby reducing oxidative stress and protecting cells from damage [16]. Moreover, Pt particles also exhibit enzyme‐like catalytic activity, enabling continuous free radical scavenging for prolonged antioxidant effects. However, despite these promising properties, Pt particles have limitations when used alone, such as poor stability and bioavailability, which restrict their practical applications. To address these challenges and enhance their therapeutic potential, Pt particles can be encapsulated in liposomes to form Pt‐liposomes. This encapsulation is expected to improve their bioactivity, stability, and skin compatibility, thereby allowing effective penetration of the skin barrier and sustained reparative effects [17, 18].

**FIGURE 1:**
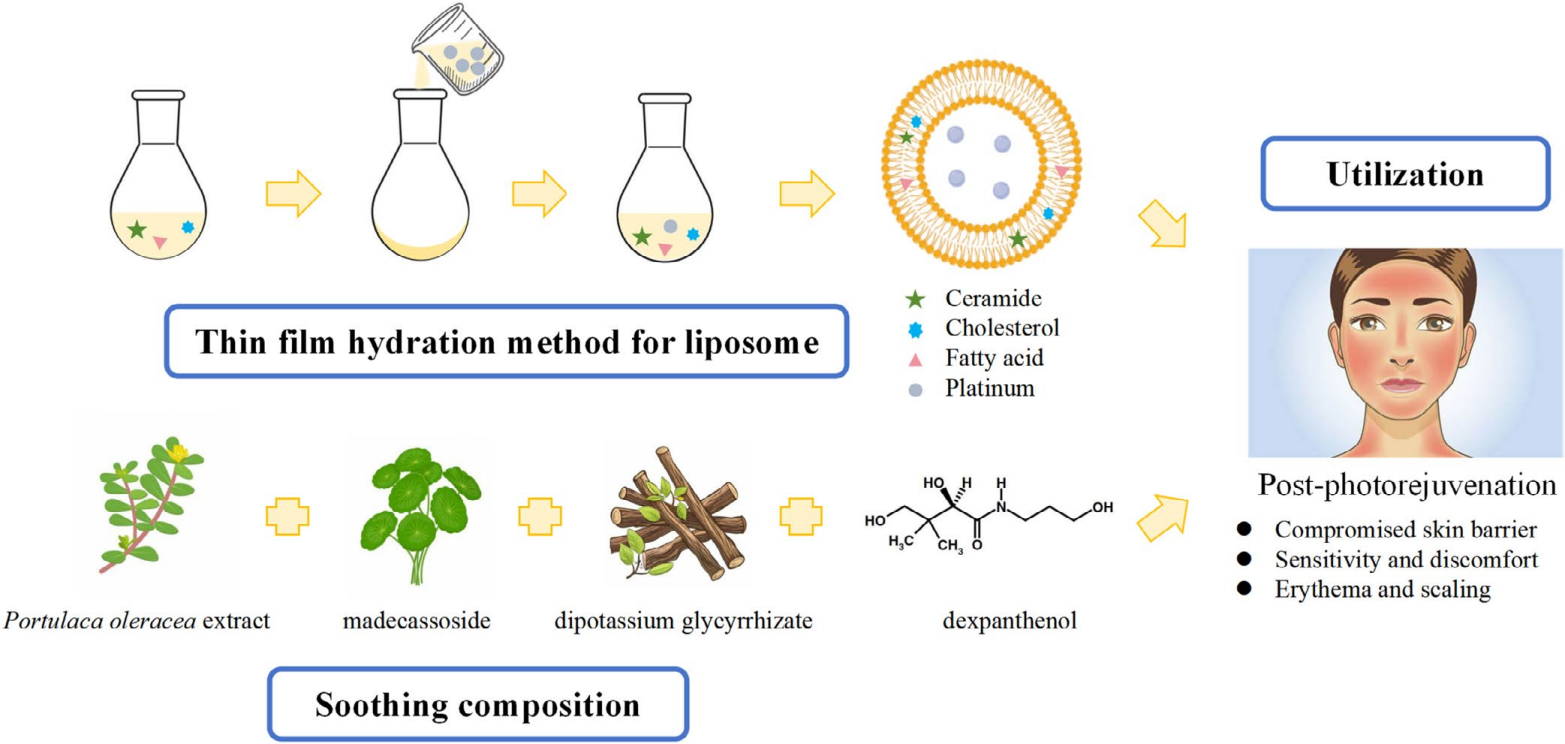
Schematic illustration of the formulation strategy and application process of the Pt‐liposome‐based facial mask.

In this study, we developed and evaluated a facial mask incorporating Pt‐liposomes, a liposomal delivery system encapsulating Pt particles, designed to enhance their stability, bioavailability, and skin penetration. To address barrier damage, erythema, and dryness following IPL photorejuvenation, the formulation combines Pt‐liposomes with key soothing agents, including dexpanthenol, dipotassium glycyrrhizate, madecassoside, and *Portulaca oleracea* extract (Figure 1). A split‐face, randomized controlled clinical trial, supported by in vitro and ex vivo analyses, was conducted to assess improvements in skin hydration, transepidermal water loss (TEWL), inflammatory responses, and key symptoms such as tightness, dryness, erythema, and scaliness during post‐photorejuvenation recovery.

## 2 Materials and Methods

### 2.1 Sample Preparation and Characterization

Pt particles were synthesized via the polyol reduction method [19] using K_2_PtCl_4_ as the precursor, with propylene glycol as the reducing agent and polyvinylpyrrolidone (PVP) as the capping agent. Pt‐liposomes were prepared using the thin‐film hydration method with a lipid composition including hydrogenated lecithin (2.5%, w/w), ceramide III B (0.55%, w/w), cholesterol (0.25%, w/w), and *Limnanthes alba* (meadowfoam) seed oil (0.15%, w/w), which was designed to mimic skin barrier lipids. The liposome formulation was further combined with a soothing composition containing panthenol, dipotassium glycyrrhizate, madecassoside, and *Portulaca oleracea* extract to create the final facial mask product. Full details on materials, formulations, and experimental procedures are provided in Appendix S1.

Particle size and dispersity of Pt particles and Pt‐liposomes were further characterized using dynamic light scattering (DLS). The encapsulation efficiency of Pt particles within liposomes was quantified by inductively coupled plasma optical emission spectrometry (ICP‐OES). Percutaneous skin penetration was evaluated through MALDI‐TOF mass spectrometry imaging (MSI), supported by histological analysis of treated skin sections. Detailed experimental procedures and conditions are summarized in Appendix S1.

### 2.2 In Vitro and Ex Vivo Evaluation of Barrier Repair and Soothing Effects

A reconstructed human epidermal model (EpiKutis) was used to evaluate the effects of Pt‐liposomes on stratum corneum morphology and lipid composition, including ceramide chain length, fatty acid levels, and cholesterol content, measured by LC–MS and HPLC. To assess potential soothing effects, histamine‐induced Ca^2+^ influx in normal human epidermal keratinocytes (NHEK) was monitored using fluorescence imaging. Anti‐inflammatory activity was evaluated in lipopolysaccharide (LPS)‐stimulated THP‐1 cells by quantifying IL‐8 mRNA expression via RT‐qPCR. Detailed experimental protocols, reagent usage, and analytical methods are provided in Appendix S1.

### 2.3 Evaluation of Skin Recovery in Irritant and Clinical Models

A sodium lauryl sulfate (SLS) patch test was performed on the forearms of four healthy volunteers to evaluate skin recovery following the application of the Pt‐liposome‐based facial mask. Recovery was assessed by measuring the erythema index (EI) and TEWL immediately after SLS‐induced irritation (D_0_) and after treatment on Days 1, 3, and 7 (D_1_, D_3_, and D_7_). The rate of change (%) was calculated using the following equation:

(1) $$D_{n}\left(\%\right)=\frac{D_{n}-{\text{D}}_{0}}{{\text{D}}_{0}}\times 100\%$$

where *D*_0_ represents the time point immediately after the modeling process, and *D*_*n*_ represents the time point *n* days after treatment with the sample.

For clinical evaluation after photorejuvenation, a randomized, split‐face trial was conducted in 30 healthy female participants aged 18 to 45. Based on visual assessment and demographic characteristics, all enrolled participants were estimated to have Fitzpatrick skin phototypes III to IV, which are typical among East Asian populations and generally exhibit moderate UV sensitivity with minimal tanning. All participants completed the study without adverse events. Ethical approval was obtained from the institutional ethics review board, and the study was registered with a national clinical trial registry. Written informed consent was obtained from all participants in accordance with the Declaration of Helsinki. Photorejuvenation was conducted by a qualified dermatologist using the Lumenis M22 intense light and laser skin treatment system. Each subject applied the Pt‐liposome‐based facial mask to one side of the face following the IPL treatment. Skin hydration, TEWL, and subjective symptoms (including tightness, dryness, erythema, and scaliness) were assessed at multiple time points over 14 days: immediately after IPL (Day 0, ad_0_), 30 min after product application on Day 1 (D_1T30min_), and on Day 3 (D_3_), Day 7 (D_7_), and Day 14 (D_14_). Note that subjective symptoms were evaluated using a standardized 10‐point questionnaire with scores ranging from 0 (no symptoms) to 9 (severe symptoms), while objective parameters were measured using a corneometer (CM825) and a TEWL analyzer (Biox AquaFlux200).

The percentage change in skin condition from baseline (BD_0_) to each time point was calculated using the following equations:

(2) $${\mathrm{AD}}_{0}\;\left(\%\right)=\frac{{\mathrm{AD}}_{0}-{\mathrm{BD}}_{0}}{{\mathrm{BD}}_{0}}\times 100\%$$

(3) $$D_{n}\left(\%\right)=\frac{D_{n}-{\mathrm{AD}}_{0}}{{\mathrm{AD}}_{0}}\times 100\%$$

where *D_n_* represents the measurement taken at each respective time point.

Detailed information on the inclusion and exclusion criteria, subject recruitment, study design, clinical procedures, outcome measurements, and statistical analysis methods for both the SLS‐induced irritant model and the photorejuvenation clinical study is provided in Appendix S1.

## 3 Results

### 3.1 Characterization of Pt Particles and Pt‐Liposomes

Pt particles were synthesized via a modified polyol reduction method using K_2_PtCl_4_ as the platinum precursor and propylene glycol as the reducing agent [19]. PVP was used as a capping agent to prevent aggregation and ensure uniform, stable dispersion of the Pt particles by forming a steric barrier on their surface. The structure and physicochemical properties of both the synthesized Pt particles and the Pt‐liposome formulation were further characterized to evaluate their size, uniformity, stability, and encapsulation performance (see Section 2 and Appendix S1 for details). DLS measurements revealed that the Pt particles had an average hydrodynamic diameter of 33.2 nm and a polydispersity index (PDI) of 0.18, indicating a relatively narrow size distribution (Figure S1, Appendix S1). Upon encapsulation into liposomes, the resulting Pt‐liposomes exhibited an average particle size of 154.5 nm with a low PDI of 0.031, suggesting a high degree of size uniformity among the Pt‐liposomes (Figure S2). The zeta potential of the Pt‐liposomes was measured at −13.15 mV, indicating moderate colloidal stability due to surface charge repulsion. The encapsulation efficiency of Pt particles within the liposomal bilayer was approximately 69.2%, as determined by inductively coupled plasma optical emission spectrometry (ICP‐OES) (see Appendix S1 for details).

### 3.2 Percutaneous Penetration of Pt‐Liposomes

To evaluate whether liposomal encapsulation enhances the transdermal delivery of Pt particles, we performed MSI of skin sections following topical application of Pt‐liposomes or unencapsulated (free) Pt particles. The imaging results are presented in Figure S3 and summarized in Table S1. Blue pixels indicate the presence of platinum (atomic mass 195 Da), with signal intensity reflecting relative Pt abundance. A time‐dependent increase in Pt‐positive pixels was observed, indicating progressive skin penetration over time. At 30 min, Pt accumulation in the Pt‐liposome group was 6.3‐fold higher than that of the Pt particle group, increasing to 14.15‐fold by 60 min.

### 3.3 Lipid Content Testing Based on 3D Epidermal Skin Model

To assess the barrier repairing effects of Pt‐liposomes, we evaluated changes in tissue morphology and epidermal lipid composition using a 3D epidermal skin model. Histological analysis revealed well‐organized, stratified epidermal structures across all groups (control, pirinixic acid, and Pt‐liposome‐treated), comprising distinct basal, spinous, granular layers, and a compact stratum corneum. Basal cells appeared intact and tightly packed, with granular layer cells showing normal differentiation (Figure S4).

**FIGURE 2:**
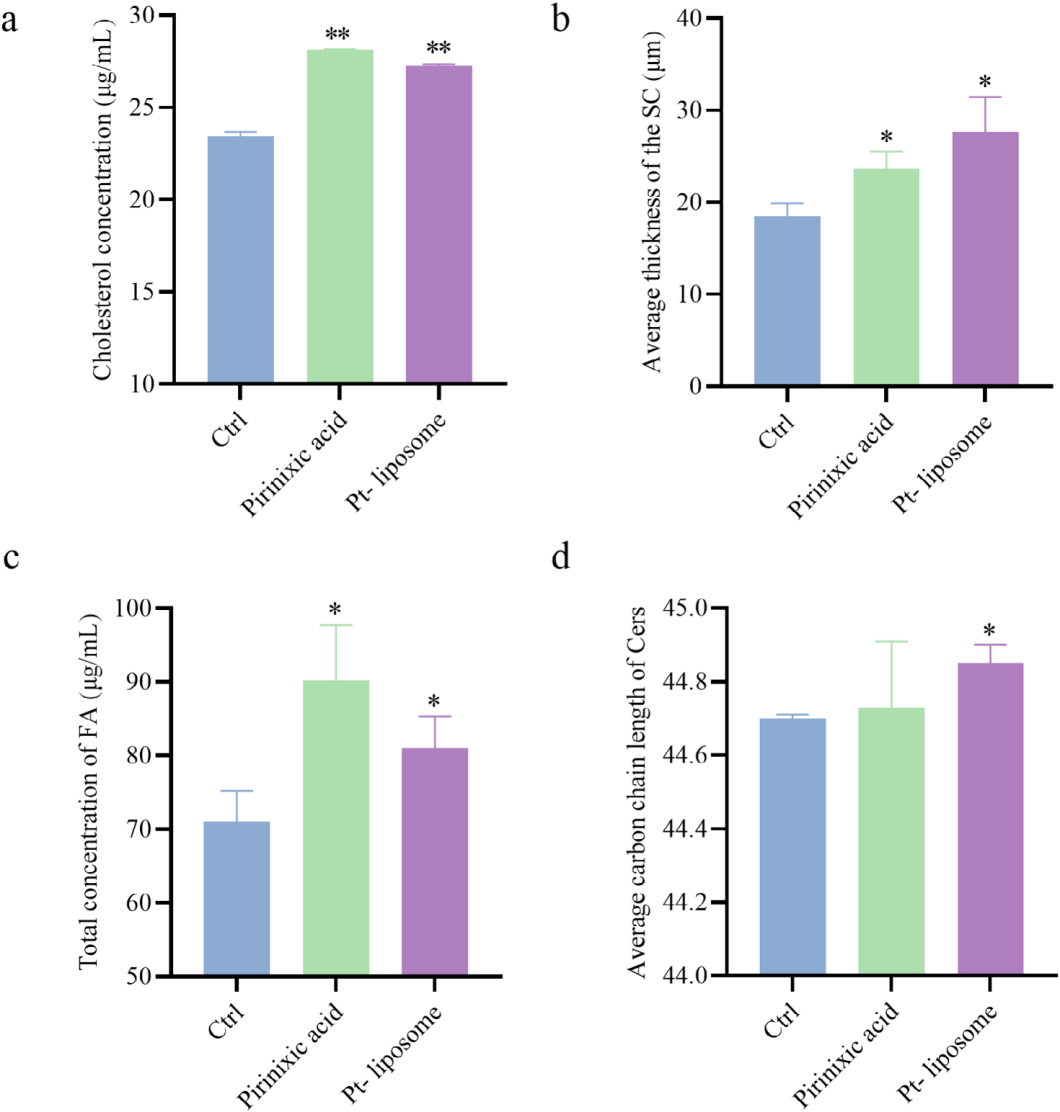
Effect of Pt‐liposomes on epidermal features in a 3D skin model. (a) Cholesterol content (*n* = 3). (b) Thickness of the stratum corneum (SC) (*n* = 3). (c) Fatty acid (FA) content (*n* = 3). (d) Carbon chain length of ceramides (Cers) (*n* = 3). Note that results are expressed as Mean ± SD. *p < 0.05 and **p < 0.01 versus the control group. Ctrl, control.

As expected, pirinixic acid, which is a known peroxisome proliferator‐activated receptor alpha (PPARα) agonist, served as an effective positive control by significantly increasing stratum corneum thickness, total fatty acid levels, and cholesterol content compared to the untreated control group. Notably, treatment with Pt‐liposomes (6.25%, v/v) resulted in an even greater increase in stratum corneum thickness, surpassing the pirinixic acid group, with enhancement rates of 49.22%. In addition, Pt‐liposomes significantly elevated the total fatty acid concentration, while cholesterol content showed a substantial increase, with enhancement rates of 14.07% and 16.26% (Figure 2a–c). In contrast, there were no significant changes in the average carbon chain length of ceramides in the stratum corneum of the skin model after treatment with pirinixic acid, compared to the control group. However, in the Pt‐liposome treated group, the average carbon chain length of ceramides in the stratum corneum was found to be significantly increased (Figure 2d).

### 3.4 Histamine Stimulation‐Calcium Imaging Test

Calcium ions (Ca^2+^) are essential intracellular messengers involved in regulating various cellular processes, including signal transduction, neurotransmitter release, and muscle contraction [20]. The concentration of intracellular calcium is tightly regulated, and any alterations in these levels serve as a key indicator of cellular responses to external stimuli. Calcium‐sensitive fluorescent dyes enable real‐time measurement of calcium fluxes, providing valuable insights into cellular behavior upon stimulation.

Histamine is a potent mediator of acute inflammation and plays a central role in immediate hypersensitivity reactions, such as allergic responses [21]. Mast cells, primarily located in the upper dermis of normal skin, release histamine in response to tissue injury or allergen exposure [22]. Once released, histamine binds to specific receptors on sensory neurons, activating unmyelinated C fibers, which transmit pruritic (itch) sensations to the central nervous system.

**FIGURE 3:**
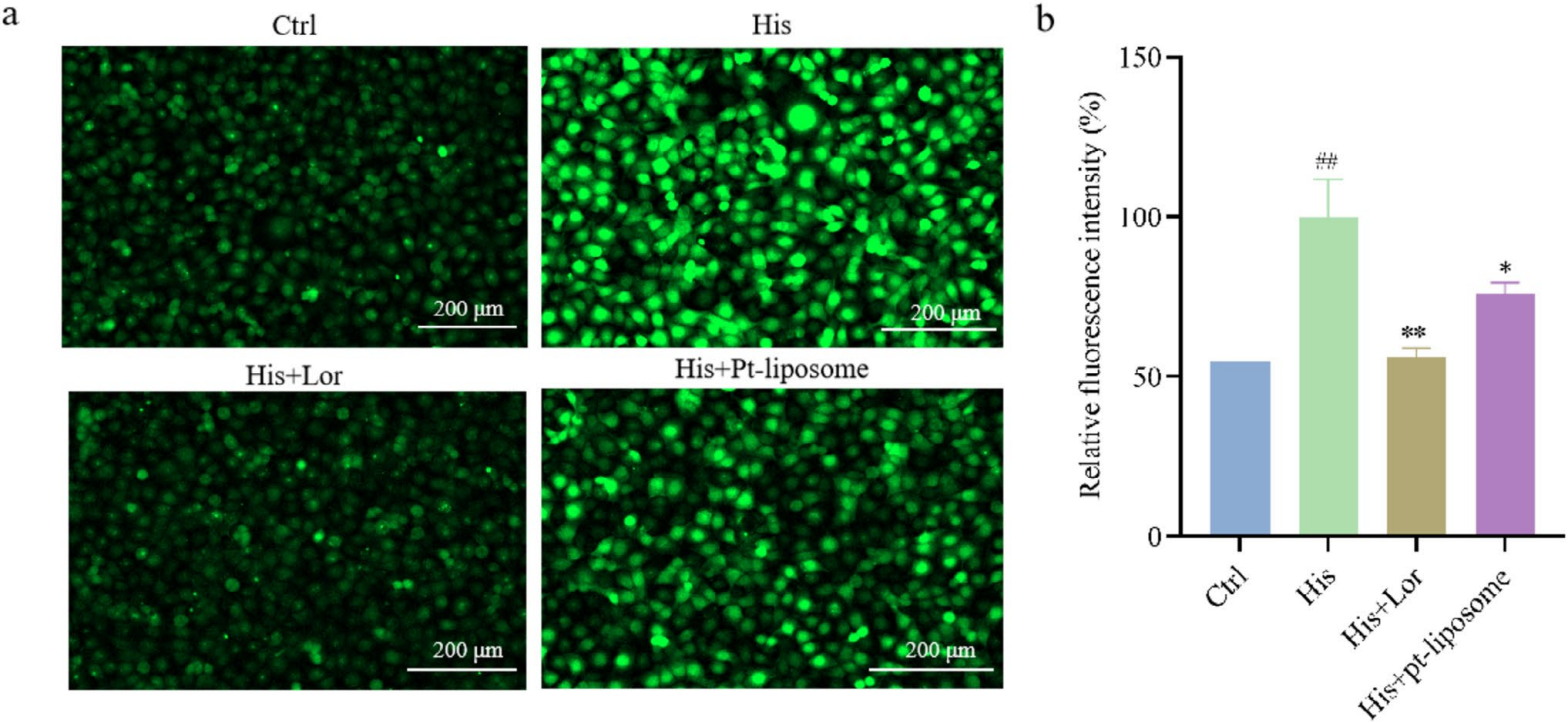
Effect of Pt‐liposomes on histamine‐induced calcium influx in NHEK. (a) Fluorescence diagram. (b) Relative fluorescence statistics. Note that results are expressed as Mean ± SD (*n* = 3). ^##^ *p* < 0.01 versus the control group. *p < 0.05 and **p < 0.01 versus the His group. Ctrl, control; His, histamine; Lor, loratadine.

In this study, intracellular calcium levels were assessed using calcium‐sensitive fluorescence imaging following histamine stimulation. Compared to the model group, the fluorescence intensity in cells treated with Pt‐liposomes (2%, v/v) was significantly reduced, indicating that Pt‐liposomes effectively inhibited the excessive calcium influx induced by histamine. The calculated inhibition rate was 24.02% (Figure 3).

### 3.5 IL‐8 mRNA Level Test

**FIGURE 4:**
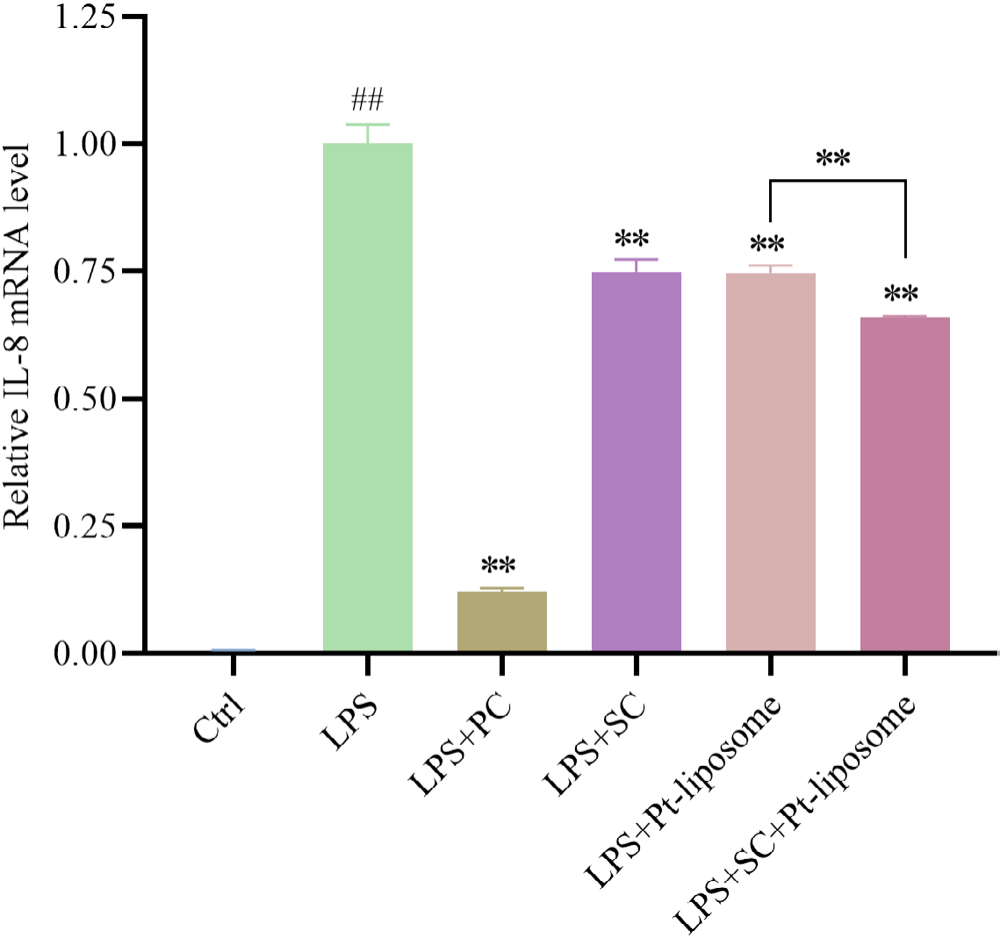
Effect of soothing composition and Pt‐liposomes on IL‐8 mRNA expression in LPS‐stimulated THP‐1 cells. Note that results are expressed as Mean ± SD (*n* = 3). ^##^ *p* < 0.01 versus the control group. **p < 0.01 versus the LPS group. Ctrl, control; LPS, lipopolysaccharide; PC, dexamethasone; SC, soothing composition.

The effects of the soothing composition and Pt‐liposomes on IL‐8 mRNA expression were further evaluated in LPS‐induced THP‐1 cells. After stimulation with lipopolysaccharide (LPS), RT‐qPCR was used to quantify IL‐8 mRNA levels in THP‐1 cells. LPS stimulation for 24 h significantly upregulated IL‐8 mRNA levels, which confirms the successful induction of inflammation. Pretreatment with either the soothing composition or Pt‐liposomes significantly reduced the LPS‐induced IL‐8 mRNA expression. Notably, the combination of 2% (v/v) soothing composition and 0.25% (v/v) Pt‐liposomes resulted in a more pronounced reduction in IL‐8 mRNA levels compared to either treatment alone, suggesting a synergistic effect (Figure 4). These results demonstrate that both the soothing composition and Pt‐liposomes can effectively inhibit LPS‐induced upregulation of IL‐8 expression, with their combination showing a more potent inhibitory effect.

### 3.6 Efficacy Test Using SLS as Irritant

The erythema index (EI) values in the test group showed a clear decrease on Day 1 (D_1_), Day 3 (D_3_), and Day 7 (D_7_), with change rates of −2.73%, −13.30%, and −17.00%, respectively. In contrast, the control group exhibited relatively modest reductions in EI values, with change rates of 0.87%, −4.51%, and −10.65% on D_1_, D_3_, and D_7_, respectively (Table S2). These results suggest that Solution A, containing the soothing composition and Pt‐liposomes, was more effective in alleviating skin redness (erythema) induced by the irritant.

Similarly, TEWL values for the test group also showed a larger decrease on D_1_, D3, and D7, with change rates of −14.88%, −41.37%, and −55.85%, respectively. In contrast, the control group exhibited smaller changes in TEWL, with rates of 11.08%, −19.59%, and −40.57% on D_1_, D_3_, and D_7_, respectively (Table S3). The more substantial decrease in TEWL values in the test group indicates improved skin barrier function, as TEWL directly measures the water retention ability and integrity of the stratum corneum. These results suggest that Solution A significantly enhances skin barrier repair and hydration, demonstrating the beneficial effects of the soothing composition and Pt‐liposomes.

### 3.7 Efficacy Test After Photorejuvenation

This study recruited 30 subjects from Guangzhou, China, all of whom met the inclusion and exclusion criteria and provided written informed consent. The study was designed as a split‐face, randomized controlled trial (RCT), ensuring the reliability and scientific rigor of the results. It is worth noting that all subjects completed the study without any dropouts. The corresponding CONSORT diagram illustrating participant flow throughout the study is shown in Figure S5.

**FIGURE 5:**
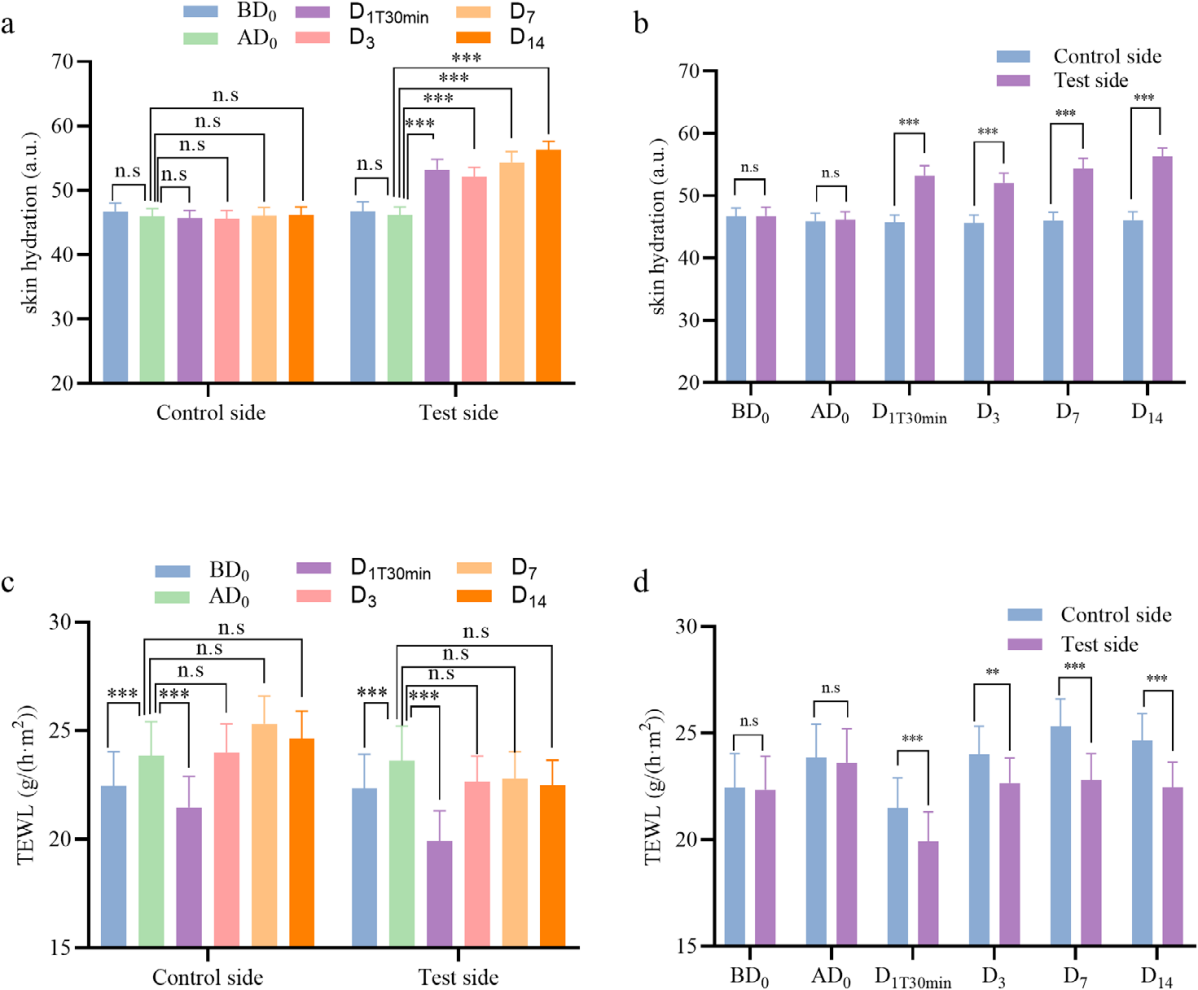
Comparison of skin hydration and transepidermal water loss (TEWL) measurements. (a) Skin hydration: Baseline (BD_0_) vs. immediately after photorejuvenation (ad_0_), and ad_0_ vs. follow‐up time points: D_1T30min_, D_3_, D_7_, and D_14_. (b) Comparison of skin hydration between the test side and control side at post‐treatment time points: D_1T30min_, D_3_, D_7_, and D_14_. (c) TEWL: BD_0_ vs. ad_0_, and ad_0_ vs. follow‐up time points: D_1T30min_, D_3_, D_7_, and D_14_. (d) Comparison of TEWL between the test side and control side at post‐treatment time points. Results are expressed as mean ± SD (*n* = 30). Statistical significance: **p < 0.01, ***p < 0.001; n.s., not statistically significant.

Skin hydration measurements were recorded on both the test and control sides of the face at baseline (BD_0_) and immediately after photorejuvenation (ad_0_). Follow‐up hydration measurements were also recorded on Day 1 (D_1T30min_), Day 3 (D_3_), Day 7 (D_7_), and Day 14 (D_14_). As shown in Figure 5a, there is no significant difference in skin hydration between BD_0_ and ad_0_ on either side of the face (*p* > 0.05). This indicates that photorejuvenation treatment, which primarily targets deeper layers of the skin to stimulate collagen production, does not affect skin hydration at the surface level. Compared to ad_0_, skin hydration was significantly increased on the test side at D_1T30min_, D_3_, D_7_, and D_14_ (*p* < 0.001), while no significant changes were observed on the control side (*p* > 0.05).

In addition, as shown in Figure 5b, there are no significant differences in skin hydration between the test and control sides at BD_0_ or ad_0_ (*p* > 0.05), further confirming that photorejuvenation alone does not influence skin hydration. However, following the application of the facial mask, skin hydration on the test side was significantly higher than on the control side at D_1T30min_, D_3_, D_7_, and D_14_ (*p* < 0.001). These results suggest that the facial mask can significantly enhance skin hydration after photorejuvenation treatment.

The TEWL measurements were taken at both baseline (BD_0_) and immediately after photorejuvenation (ad_0_). Additional TEWL results were obtained at various follow‐up time points. As shown in Figure 5c, TEWL values increased significantly on both sides at ad_0_ compared to BD_0_ (*p* < 0.001). This indicates that photorejuvenation may temporarily disrupt the skin barrier and induce micro‐damage to the stratum corneum, leading to increased water loss through the epidermis. Note that such impairment of the skin barrier function is a commonly observed response after photorejuvenation treatments. Compared to ad_0_, both the test and control sides showed significantly decreased TEWL at D_1T30min_ (*p* < 0.001), but no significant differences were observed at later time points, that is, D_3_, D_7_, and D_14_ (*p* > 0.05).

As shown in Figure 5d, there is no significant difference in TEWL values between the test and control sides at either BD_0_ or ad_0_ (*p* > 0.05), suggesting that photorejuvenation does not immediately influence TEWL when comparing these two sides. However, after photorejuvenation, a more pronounced reduction in TEWL was observed on the test side compared to the control side on Day 1 (D_1T30min_), Day 3 (D_3_), Day 7 (D_7_), and Day 14 (D_14_) (*p* < 0.01). This indicates that the test sample significantly promotes skin repair, restoring the integrity of the skin barrier and improving its ability to retain moisture.

As shown in Figure S6, there were no significant differences in skin tightness, dryness, or scaliness between baseline (BD_0_) and immediately after photorejuvenation (ad_0_) (*p* > 0.05). However, erythema severity significantly increased immediately following photorejuvenation (*p* < 0.001), indicating that the treatment induced acute inflammation. Compared to ad_0_, the test side showed significant improvements in skin tightness, erythema, dryness, and scaliness at D_1T30min_, D_3_, D_7_, and D_14_. In contrast, the control side exhibited improvements in skin tightness and dryness at D_7_ and D_14_, although the magnitude of improvement was lower than that observed on the test side. The control side also demonstrated significant reductions in erythema at D_1T30min_, D_3_, D_7_, and D_14_, but to a lesser extent than the test side. Notably, no improvement in scaliness was observed on the control side at any time point.

**FIGURE 6:**
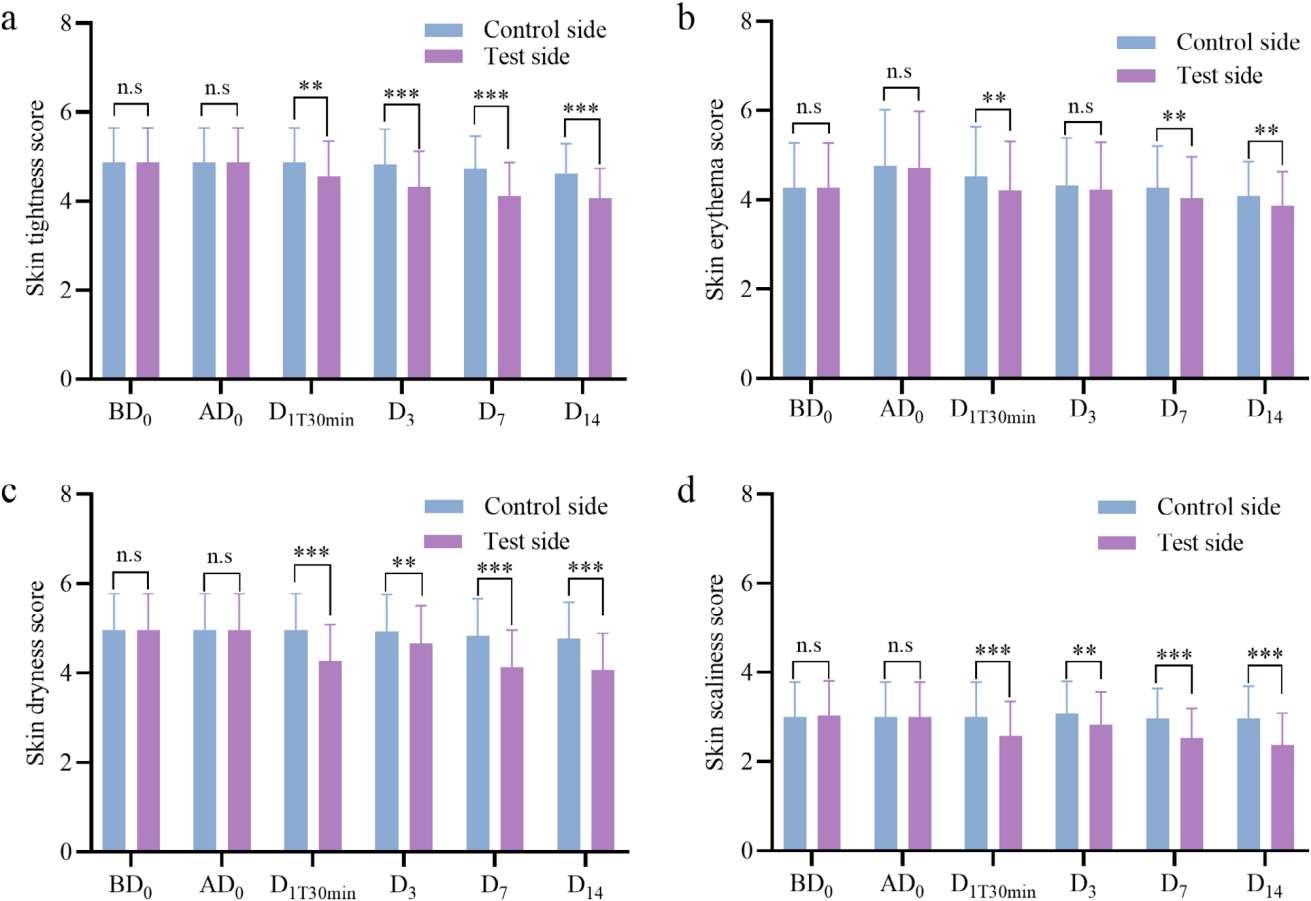
Subjective assessment scores for skin conditions. (a) Skin tightness score from researcher. (b) Skin erythema score from researcher. (c) Skin dryness score from researcher. (d) Skin scaliness score from researcher. Note that results are expressed as mean ± SD (*n* = 30). **p < 0.01 and ***p < 0.001. n.s (not statistically significant).

As shown in Figure 6b, the degree of erythema was significantly more improved on the test side than on the control side on Day 1 (D_1T30min_) after photorejuvenation treatment (*p* < 0.01). However, no significant difference in erythema was observed between the test and control sides on Day 3 (D_3_) (*p* > 0.05). One possible explanation for this is that the skin experienced immediate relief following the application of the facial mask, but values converged by Day 3 as the natural recovery and self‐repair mechanisms of the skin took effect. The degree of erythema continued to improve significantly on the test side compared to the control side on Day 7 (D_7_) and Day 14 (D_14_) after the treatment (*p* < 0.01), indicating that the effect of the facial mask is sustained over time. As shown in Figure 6a,c,d, the degree of skin tightness, dryness, and scaliness was significantly more improved on the test side than on the control side on D_1T30min_, D_3_, D_7_, and D_14_ after photorejuvenation treatment (*p* < 0.01). This indicates that the facial mask can effectively alleviate skin tightness, dryness, and scaliness following photorejuvenation.

**FIGURE 7:**
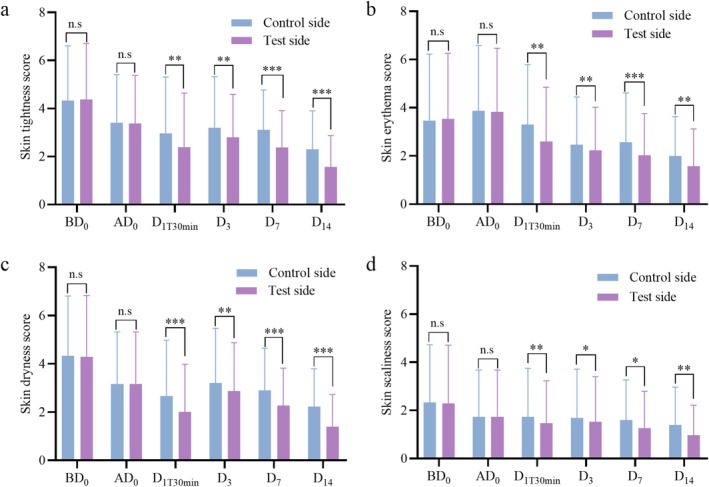
Self‐assessment Scores from subjects. (a) Skin tightness score from subjects. (b) Skin erythema score from subjects. (c) Skin dryness score from subjects. (d) Skin scaliness score from subjects. Note that results are expressed as mean ± SD (*n* = 30). *p < 0.05, **p < 0.01, and ***p < 0.001. n.s (not statistically significant).

As summarized in Figure 7a–d, no significant differences were found in skin tightness, erythema, dryness, or scaliness between the test and control sides on BD_0_ or ad_0_ (*p* > 0.05). Self‐reported assessments by the subjects indicated that the degree of skin tightness, erythema, dryness, and scaliness was more improved on the test side than on the control side on D_1T30min_, D_3_, D_7_, and D_14_ (*p* < 0.05). This aligns with the dermatological assessment, further suggesting that the facial mask has a positive impact on skin condition following photorejuvenation. It is worth noting that under the conditions of this study, subjects applied the facial mask for 14 days following photorejuvenation treatment. According to the dermatological assessment, there were no adverse reactions related to the test sample. Additionally, 100% of the subjects reported no irritation or discomfort after using the facial mask, and all volunteers (100%) expressed satisfaction with the test sample.

## 4 Discussion

The results of this study highlight the potential of Pt‐liposomes as a reparative and soothing topical ingredient for skin barrier restoration. Using the thin‐film hydration method, we successfully prepared Pt‐liposomes with uniform size, good colloidal stability, and high platinum encapsulation efficiency. Time‐dependent increases in platinum signal intensity within skin tissue confirmed enhanced percutaneous penetration, suggesting deposition into viable epidermal layers where lipid synthesis and remodeling occur.

It is well known that the stratum corneum relies on a highly organized lipid matrix to maintain barrier integrity, which is primarily composed of cholesterol, fatty acids, and ceramides. Disruptions in these lipids, particularly reductions in long‐chain ceramides and cholesterol, are linked to increased TEWL and heightened skin sensitivity. In this study, Pt‐liposome treatment increased stratum corneum thickness, elevated cholesterol and fatty acid levels, and extended average ceramide chain length, indicating enhanced lipid organization and improved barrier repair.

Functionally, Pt‐liposomes exhibit both barrier restorative and anti‐inflammatory properties. Their lipid bilayer, comprising ceramides, cholesterol, and *Limnanthes alba* (meadowfoam) seed oil, closely mimics the physiological composition of stratum corneum lipids and likely aids in replenishing lipid components lost during Pt delivery. At the same time, the encapsulated platinum nanoparticles catalyze redox reactions, offering potent antioxidative and anti‐inflammatory effects by restoring cellular redox balance, which is a key mechanism for protecting skin cells against oxidative stress and inflammation [10, 17, 23]. The combined action of these components supports both lipid barrier restoration and suppression of inflammatory responses. As evidenced by the histamine‐induced calcium influx assay, Pt‐liposomes significantly inhibited excessive calcium entry, suggesting a role in modulating cutaneous nerve hyperresponsiveness.

**FIGURE 8:**
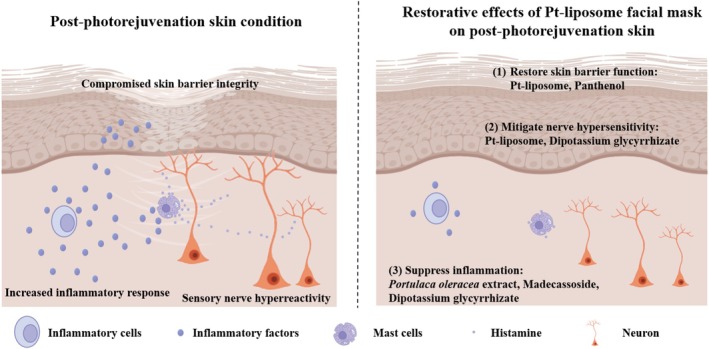
Schematic illustration of how the Pt‐liposome‐based facial mask helps alleviate sensitive skin symptoms.

Furthermore, when combined with soothing agents, including panthenol, dipotassium glycyrrhizate, madecassoside, and *Portulaca oleracea* extract, the Pt‐liposome‐based formulation showed a synergistic anti‐inflammatory effect, as evidenced by the significant reduction in IL‐8 mRNA expression. Each soothing agent plays a complementary role in mitigating inflammation and enhancing skin recovery. For example, panthenol supports hydration and epidermal repair by promoting keratinocyte differentiation and improving lipid lamellae flexibility, especially under dry conditions [24, 25, 26]. Dipotassium glycyrrhizate inhibits histamine release and hyaluronidase activity, alleviating itching and allergic responses [27, 28, 29]. Madecassoside can modulate inflammation by downregulating cytokine expression and suppressing NF‐κB, and COX‐2 and iNOS signaling pathways [30, 31]. *Portulaca oleracea* extract has immunomodulatory properties, enhancing the activity of natural killer (NK) cells and T lymphocytes. It also helps regulate the balance of Th1/Th2 cytokines, including IL‐4, IL‐10, IFN‐γ, and TNF‐α, which supports skin immune homeostasis and reduces inflammation in sensitive skin [32] (Figure 8).

Photorejuvenation was found to acutely disrupt skin barrier function, as indicated by increased TEWL and erythema, without changes in hydration. This suggests increased skin fragility and reactivity post‐treatment. As such, post‐procedure care must address impaired barrier function, nerve hyperresponsiveness, and inflammation. Our clinical data confirmed the benefits of the Pt‐liposome‐based facial mask in mitigating these issues. Improvements in skin hydration, tightness, erythema, dryness, and scaliness were consistently observed on the test side compared to the control, particularly during the critical 14‐day post‐treatment period. These findings support the efficacy of the Pt‐liposome‐based mask in accelerating skin recovery and restoring barrier function after photorejuvenation. Moreover, no adverse effects were observed during the study, confirming that the formulation is both effective and well‐tolerated.

It is important to note that while our findings demonstrate short‐term benefits, particularly in enhancing lipid profiles and modulating inflammation, the long‐term durability of these effects remains unknown and is beyond the scope of this study. Future time‐course studies will be necessary to evaluate the sustained impact of Pt‐liposome treatment on skin barrier function and overall skin health. In addition, although our in vitro analyses demonstrated the individual contributions of Pt‐liposomes and soothing agents, the clinical study evaluated their combined formulation. As such, the observed in vivo outcomes reflect the integrated effects of all active components under real‐world conditions, limiting attribution to specific ingredients. Future studies should aim to include component‐specific clinical evaluations to better clarify their respective contributions.

To contextualize our findings, we compared our study with previous reports on postoperative skincare strategies following IPL or laser skin resurfacing (LSR) treatments [33, 34, 35] (Table 1). While many earlier studies focused on antioxidant serums, botanical extracts, or mineral sunscreens to reduce redness and discomfort [33, 34], few have systematically combined barrier restoring lipids with anti‐inflammatory and antioxidative agents within a controlled clinical study. Our Pt‐liposome formulation integrates ceramides, cholesterol, hydrogenated lecithin, *Limnanthes alba* (meadowfoam) seed oil, and platinum particles into a novel delivery system. Its functionality was further enhanced by combining Pt‐liposomes with established soothing agents, including panthenol, dexpanthenol, dipotassium glycyrrhizate, madecassoside, and *Portulaca oleracea* extract, designed to amplify both anti‐inflammatory and barrier‐repairing effects. Compared to prior approaches, our study offers new clinical evidence supporting improvements in skin hydration, TEWL reduction, and erythema relief, highlighting the potential of this formulation as a multifunctional post‐photorejuvenation treatment.

**TABLE 1:** Comparison of the present study with previous reports on skincare strategies following esthetic treatments.

Research objective	Esthetic treatment	Post‐treatment intervention	Outcome			Ref.
			Skin hydration	Skin barrier repair	Erythema	
To evaluate the efficacy of a Pt‐liposome‐based facial mask containing anti‐inflammatory and barrier‐restoring ingredients after IPL treatment	Intense pulsed light (IPL)	A Pt‐liposome‐based mask containing Pt‐liposomes, dexpanthenol, dipotassium glycyrrhizate, madecassoside, and *Portulaca oleracea* extract. The Pt‐liposomes consist of Pt particles, ceramides, cholesterol, hydrogenated lecithin, and *Limnanthes alba* seed oil.	Methods: Clinical study with instrumental analysis. Results: Noticeable improvement in hydration at Day 1 (30 min post‐treatment), Day 3, Day 7, and Day 14 compared to control (*p* < 0.001).	Methods: Clinical study with instrumental analysis. Results: Noticeable reduction in TEWL at Day 1 (30 min post‐treatment), Day 3, Day 7, and Day 14 compared to control (*p* < 0.01).	Methods: Subjective assessment. Results: Erythema severity significantly decreased on the test side compared to the control side at Day 7 and Day 14 (*p* < 0.01).	This work
To evaluate the efficacy and tolerability of combining a topical skincare regimen (TSCR) with a single IPL treatment for mild‐to‐severe facial redness associated with rosacea	IPL	A 3‐in‐1 facial cream with vitamins, herbal ingredients, and SPF 50 mineral sunscreen (titanium dioxide 11.6%, zinc oxide 8.6%).	N.A.	N.A.	Methods: Subjective assessment. Results: Using a 7‐point redness scale, the mean (SD) redness score noticeable improved from 3.05 (0.97) at baseline to 2.05 (0.76) at Week 18 (*p* < 0.01).	[33]
To evaluate whether a phyto‐corrective mask, gel, and resveratrol antioxidant serum improve outcomes after IPL treatment	IPL	Phyto‐corrective mask, gel, and resveratrol BE serum	Methods: Subjective assessment. Results: Noticeable improvements in hydration were observed on the treated side at Day 1, 1 month, and 3 months post‐treatment.	N.A.	Methods: Subjective assessment. Results: Noticeable improvements in erythema and overall skin appearance were observed on the treated side at Day 1, 1 month, and 3 months post‐treatment.	[34]
To evaluate *Centella asiatica* extract on wound healing after laser resurfacing	Laser skin resurfacing (LSR)	0.05% w/w ECa 233 gel	N.A.	N.A.	Methods: Clinical study with instrumental analysis. Results: The side treated with 0.05% w/w ECa 233 gel showed a significantly lower erythema index throughout the follow‐up period, with a reduction of 0.03 U.	[35]

## 5 Conclusion

In this study, we demonstrated that platinum (Pt)‐liposomes can have significant soothing and reparative effects, evidenced through lipid content analyses in a 3D epidermal skin model and histamine stimulation‐calcium imaging assays. It was found that Pt‐liposomes effectively inhibited histamine‐induced calcium influx by 24.02%, highlighting their ability to reduce cutaneous hypersensitivity. When combined with a soothing composition containing panthenol, dipotassium glycyrrhizate, madecassoside, and *Portulaca oleracea* extract, the Pt‐liposome‐based formulation exhibited a potent anti‐inflammatory effect, significantly reducing IL‐8 mRNA expression by 45.8% compared to controls. Clinical evaluations in a split‐face randomized trial further supported these findings, revealing that a facial mask infused with Pt‐liposomes enhanced skin hydration by 48.6% on Day 14 (*p* < 0.001) and reduced TEWL by 55.85% on Day 7 (*p* < 0.01), indicating a significant recovery of skin barrier function. Subjective assessments further showed substantial improvements in erythema, tightness, dryness, and scaliness on the treated side at Day 1, Day 3, Day 7, and Day 14 after photorejuvenation (*p* < 0.01), including a 36.7% reduction in erythema by Day 7. Importantly, the treatment was well‐tolerated with no adverse reactions reported, affirming its safety and efficacy for sensitive skin post‐photorejuvenation. Overall, this study provides robust clinical evidence supporting Pt‐liposome‐based formulations as an effective therapeutic strategy for accelerating skin barrier repair, reducing inflammation, and promoting both rapid soothing and long‐term skin health improvement following photorejuvenation treatments.

## Author Contributions

**Zhixin Du:** conceptualization, investigation, writing – original draft, writing – review and editing, visualization. **Lu Ren:** conceptualization, methodology, writing – review and editing. **Hui Liang:** formal analysis, data curation, writing – original draft, writing – review and editing. **Yongjie Lu:** formal analysis, data curation, writing – original draft, writing – review and editing. **Dongying Zhang:** formal analysis, data curation, writing – original draft, writing – review and editing. **Zhanghao Li:** visualization, writing – review and editing. **Shuangyan Wang:** formal analysis, data curation, writing – review and editing. **Kangjin Zhang:** formal analysis, data curation, writing – review and editing. **Ying Wu:** visualization, writing – review and editing. **Dongcui Li:** conceptualization, project administration, supervision, writing – review and editing. **Li Ye:** conceptualization, project administration, supervision, writing – review and editing. **Naisheng Jiang:** conceptualization, supervision, writing – review and editing.

## Ethics Statement

This study has obtained approval from the Ethics Committee of the Dermatology Hospital of Southern Medical University, with an approval date of May 6, 2024, and an approval number of 2024036. Additionally, this research has been registered at the China Clinical Research Center, with a registration number of ChiCTR2400091907. Signed informed consent forms were obtained from each participant. This study was conducted in accordance with the Helsinki Declaration.

## Conflicts of Interest

Authors Zhixin Du, Lu Ren, Hui Liang, Yongjie Lu, Dongying Zhang, Zhanghao Li, Shuangyan Wang, Kangjin Zhang, and Dongcui Li were employed by the company Hua An Tang Biotech Group Co. Ltd. The other authors declare no conflicts of interest.

## Supporting information


**Figure S1:** Size distribution of Pt particles measured by DLS.
**Figure S2:**. Size distribution of Pt‐liposomes measured by DLS.
**Figure S3:**. Mass spectrometry imaging (MSI) of mouse skin sections following topical application of Pt particles or Pt‐liposomes for 10, 30, or 60 min, respectively. Pixel intensity maps depict the spatial distribution and relative abundance of platinum (Pt, 195 Da) within the tissue.
**Figure S4:**. Histomorphometric analysis of 3D epidermal skin models after 24 h incubation with control, pirinixic acid, or Pt‐liposome. Tissues were fixed in 4% paraformaldehyde for 24 h and stained with hematoxylin and eosin (H&E) for morphological evaluation.
**Figure S5:**. Flowchart of inclusion, randomization, and participant progression throughout the study.
**Figure S6:**. Subjective skin evaluation scores before and after photorejuvenation treatment. (a) Skin tightness score. (b) Skin erythema score. (c) Skin dryness score. (d) Skin scaliness score. Results are expressed as mean ± SD. Statistical significance: *p < 0.05, **p < 0.01, ***p < 0.001; n.s., not statistically significant.
**Table S1:** Relative distribution of platinum (Pt) in skin tissue at different time points following topical application.
**Table S2:** Changes in erythema index (EI) values at different time points in the control and test groups (mean ± SD).
**Table S3:** Changes in transepidermal water loss (TEWL) values at different time points in the control and test groups (mean ± SD).

## Data Availability

The data that support the findings of this study are available on request from the corresponding author. The data are not publicly available due to privacy or ethical restrictions.
