# Supplementary material for: A Split‐Face Randomized Study on the Efficacy of a Platinum‐Liposome‐Based Facial Mask Containing Soothing Ingredients for Post‐Photorejuvenation Skin Recovery
Source: J Cosmet Dermatol. 2025 Sep 16;24(9):e70452. doi: 10.1111/jocd.70452 (PMC12440843; doi:10.1111/jocd.70452)
Supplement: Supplementary file 1 — Figure S1: Size distribution of Pt particles measured by DLS. Figure S2:. Size distribution of Pt‐liposomes measured by DLS. Figure S3:. Mass spectrometry imaging (MSI) of mouse skin sections following topical application of Pt particles or Pt‐liposomes for 10, 30, or 60 min, respectively. Pixel intensity maps depict the spatial distribution and relative abundance of platinum (Pt, 195 Da) within the tissue. Figure S4:. Histomorphometric analysis of 3D epidermal skin models after 24 h incubation with control, pirinixic acid, or Pt‐liposome. Tissues were fixed in 4% paraformaldehyde for 24 h and stained with hematoxylin and eosin (H&E) for morphological evaluation. Figure S5:. Flowchart of inclusion, randomization, and participant progression throughout the study. Figure S6:. Subjective skin evaluation scores before and after photorejuvenation treatment. (a) Skin tightness score. (b) Skin erythema score. (c) Skin dryness score. (d) Skin scaliness score. Results are expressed as mean ± SD. Statistical significance: *p < 0.05, **p < 0.01, ***p < 0.001; n.s., not statistically significant. Table S1: Relative distribution of platinum (Pt) in skin tissue at different time points following topical application. Table S2: Changes in erythema index (EI) values at different time points in the control and test groups (mean ± SD). Table S3: Changes in transepidermal water loss (TEWL) values at different time points in the control and test groups (mean ± SD). [file JOCD-24-e70452-s001.docx]

**Supporting Information for**

**A Split-Face Randomized Study on the Efficacy of a Platinum-Liposome-Based Facial Mask Containing Soothing Ingredients for Post-Photorejuvenation Skin Recovery**

**Additional Materials and methods**

**1.1 Materials**

The following materials were used in this study: EpiGrowth cultured liquid was obtained from Guangdong Biocell Biotechnology Co., Ltd., and PBS from Solarbio. Polyoxymethylene was sourced from Biosharp, and Pirinixic Acid was purchased from Sigma. For the preparation of solutions, methanol, isopropyl alcohol, and trichloromethane were supplied by Sigma and Sinopharm, respectively. Normal human epidermal keratinocytes (NHEK) were obtained from BNCC, and EMEM was also sourced from BNCC. Other chemicals and reagents included loratadine (MCE), Fluo-4/AM (Beyotime), histamine (MCE), and RPMI-1640 complete medium (containing 10% FBS) from BeNa Culture Collection. Ceramide III B (Purity: 95%) ware purchased from Evonik Industries AG. Potassium tetrachloroplatinate (II) (K_2_PtCl_4_) was purchased from Macklin (Shanghai, China), propylene glycol (PG) was purchased from DuPont Tate & Lyle (USA) and polyvinylpyrrolidone (PVP) was purchased from Sigma-Aldrich (Shanghai, China).

For cell culture and experiments, lipopolysaccharide (Sigma), dexamethasone (Sigma), and THP-1 cells (China Center for Type Culture Collection) were used. Primers for IL-8 and GAPDH were purchased from Beijing Rui Biotech Co., Ltd. Additionally, penicillin-streptomycin solution (Gibco), HiScript II Q RT SuperMix for qPCR (+gDNA wiper) (Vazyme), and RaPure Total RNA Micro Kit (Magen) were used for RNA isolation and gene expression analysis. PowerUp™ SYBR™ Green Master Mix from Thermo Fisher Scientific was employed for qPCR analysis.

Other reagents and materials used included 75% alcohol from Shandong Lierkang Medical Technology Co., Ltd., sodium lauryl sulfate solution from Tianjin Damao Chemical Reagent Factory, and a spot tester (diameter 18 mm, depth 1.2 mm) from Beijing Billion Yida Technology Development Co., Ltd. Medical dressings were provided by the Dermatology Hospital of Southern Medical University for clinical evaluations.

**1.2 Sample preparation**

Pt particles were synthesized via the polyol reduction method^19^. Herein, K_2_PtCl_4_ was reduced by propylene glycol (PG) at 110 ℃ and then Pt particles were formed and capped by polyvinylpyrrolidone (PVP). Pt particles solution with 2000 ppm content of Pt was obtained for further use after multiple times washing and redispersing of Pt particles with water.

To prepare the Pt-liposome, a thin-film hydration method was employed. Hydrogenated lecithin (2.5%, w/w), ceramide III B (0.55%, w/w), cholesterol (0.25%, w/w), and *Limnanthes alba* (meadowfoam) seed oil (0.15%, w/w) were blended and dissolved in a mixed organic solvent of methanol and dichloromethane. Ceramide III B was selected for its structural similarity to natural human ceramides and its ability to restore lamellar lipid bilayers in the stratum corneum. This solution was then evaporated at 65 °C using a rotary evaporator to remove the organic solvent, forming a dry film. Pt particles solution (8%, w/w) and 1,3-butanediol were added to rehydrate the film and form a liposomal suspension. The suspension was homogenized using a high-pressure homogenizer to form uniform Pt-liposome.

For the soothing composition, panthenol (5%, w/w), dipotassium glycyrrhizate (0.1%, w/w), madecassoside (0.01%, w/w), and Portulaca oleracea extract (0.015%, w/w) were mixed in specific proportions. Solution A was prepared by combining Pt-liposome (0.05%, w/w), soothing composition (5.125%, w/w), water, a small amount of thickener, and preservative. Solution B consisted of water, a small amount of thickener, and preservative. To prepare the facial mask, Solution A was applied to a facial mask cloth. Finally, the moisturizing lotion was made by combining a small amount of moisturizer into a skin care lotion formulation.

**1.3 Dynamic light scattering (DLS) measurements**

DLS was employed to measure the hydrated particle size of Pt particles and Pt-liposome with the Malvern Zetasizer Advance instrument. Pt particles and Pt-liposome were diluted with pure water to the appropriate concentration for the measurement.

**1.4 Encapsulation efficiency of Pt-liposome**

To determine the encapsulation efficiency of Pt particles within the liposomes, the Pt-liposome suspension was subjected to ultracentrifugation using a 10 kDa molecular weight cut-off membrane to separate unencapsulated (free) Pt particles from the liposome-encapsulated fraction. The platinum content in both the supernatant (free Pt) and the total Pt-liposome suspension was quantified using inductively coupled plasma optical emission spectrometry (ICP-OES). The encapsulation efficiency (EE) was calculated using the following equation:

$$\text{Encapsulation efficiency (\%) = }\left( 1-\frac{\text{M}_{\text{free Pt}}}{\text{M}_{\text{total Pt}}} \right)\times100\% \text{ (eq. S1)}$$

where 𝑀_free Pt_ is the amount of unencapsulated (free) Pt in the supernatant obtained after ultracentrifugation, and 𝑀_total Pt_ is the total amount of Pt in the original Pt-liposome suspension before ultracentrifugation.

**1.5 In vivo percutaneous penetration test**

Six-week-old female nude mice were acclimatized for one week and randomly assigned to three groups: (1) control (propylene glycol), (2) Pt particles, and (3) Pt-liposome. A total of 200 μL of each sample was topically applied to the shaved dorsal skin of the mice. For time-course evaluation, the treatment was administered as follows: 10 min (single application), 30 min (three applications at 10-min intervals), and 60 min (six applications at 10-min intervals). At the end of each treatment period, mice were euthanized, and skin samples from the application site (2 cm × 2 cm) were collected and fixed in 4% paraformaldehyde for 24 h.

The treated skin was processed for both cryosectioning and paraffin sectioning. Cryosections were subjected to matrix-assisted laser desorption/ionization time-of-flight mass spectrometry (MALDI-TOF-MS) to visualize platinum penetration by detecting the elemental mass of Pt (195 Da). MSI analysis was performed in reflector negative ion mode with a raster width of 100 μm and 500 laser shots per pixel. Data acquisition, preprocessing, and visualization were performed using the Bruker Daltonics FLEX software package, including FlexControl 3.4, FlexAnalysis 3.4, and FlexImaging 3.0. Spectral data were processed using a Gaussian smoothing algorithm (0.02 m/z width, 2 cycles). In parallel, paraffin-embedded sections were stained with hematoxylin and eosin (H&E) and examined microscopically to assess skin tissue morphology. Imaging data from MALDI-TOF-MS and histological observations were co-analyzed for spatial correlation and qualitative evaluation of Pt penetration.

**1.6 Lipid content assessment using a 3D epidermal skin model**

The lipid-regulating efficacy of the test samples was evaluated using a 3D epidermal skin model (EpiKutis, batch ES221206; Guangdong Biocell Biotechnology Co., Ltd.). Models were assigned to three groups: blank control (Control), positive control (Pirinixic Acid), and sample group. Assessments included tissue morphology and quantification of ceramides, fatty acids, and cholesterol following treatment.

For sample preparation, a 50 μM working solution of Pirinixic Acid was prepared by dissolving 10 μL of a 30 mM stock solution in 6 mL of culture medium. The models were placed into a 6-well plate according to the assigned groups, ensuring proper labeling of each test group. After treatment, the models were incubated in a CO_2_ incubator at 37 °C with 5% CO_2_ for 24 h. After incubation, the models were washed with sterile PBS to remove any residual substances. Sterile cotton swabs were used to wipe any excess liquid from both the interior and exterior of the models.

The tissue morphology of the models was assessed by excising the samples and fixing them in 4% paraformaldehyde for 24 h. After fixation, the samples underwent H&E staining, and tissue morphology was observed microscopically. Images were captured for further analysis. For testing ceramides and fatty acids, the models were placed into a centrifuge tube containing proteinase K solution and incubated in a 50 °C water bath for 2 h. The stratum corneum was rinsed with deionized water using tweezers and transferred to a new centrifuge tube. Lipid extraction was carried out using a methanol:chloroform (1:2) solution, followed by sonication for 2 min in an ice-water bath. The extraction was repeated with a methanol:chloroform (2:1) solution. After extraction, the solution was dried under nitrogen, and the dried extract was resuspended in isopropanol. The samples were centrifuged at 14,000 rpm for 10 min, and the supernatant was collected for LC-MS analysis.

For cholesterol testing, the models were cut into smaller pieces and placed into 1.5 mL centrifuge tubes. Each tube received 500 μL of 0.2 mg/mL proteinase K solution. The tubes were incubated in a 50 °C water bath for 1 h. After incubation, 250 μL of methanol was added, and the samples underwent sonication for 30 min. Following sonication, the samples were centrifuged at 14,000 rpm for 10 min, and the methanol was evaporated at 60°C. The dried samples were stored at 4 °C for further analysis using HPLC.

GraphPad Prism was used to generate graphs. Data (n = 3) are expressed as mean ± SD. Statistical comparisons between groups were performed using two-tailed t-tests, with *p* < 0.05 considered statistically significant and *p* < 0.01 highly significant. Graphs were generated using GraphPad Prism.

**1.7 Histamine stimulation-calcium imaging test**

Normal human epidermal keratinocytes (NHEK) were cultured in EMEM supplemented with 10% FBS at 37°C in a 5% CO_2_ atmosphere. Cells were seeded in a 96-well plate and incubated for 24 h. After a 3 h pre-treatment with either the positive control (loratadine) or Pt-liposome, the culture medium was removed, and the cells were incubated with HBSS containing Fluo-4 AM for 45 min at 37 °C. Following Fluo-4 AM loading, the cells were washed 5 times with HBSS and pretreated with loratadine or Pt-liposome for an additional 30 min. Kinetic calcium imaging was performed using a Biotek Cytation cell imaging multifunctional microplate reader. Following a 1 min baseline recording, 10 µM histamine was added to stimulate the cells. The maximum relative fluorescence intensity was recorded 2 min after histamine stimulation. Lower fluorescence intensity indicated greater inhibition of histamine-induced calcium influx, reflecting a stronger soothing effect.

GraphPad Prism was used to generate graphs, with results expressed as mean ± SD (n = 3). Comparisons between groups were performed using two-tailed t-tests, with *p* < 0.05 considered statistically significant and *p* < 0.01 highly significant.

**1.8 IL-8 mRNA level test**

THP-1 cells were cultured in RPMI-1640 complete medium supplemented with 10% FBS, 100 U/mL penicillin, and 100 U/mL streptomycin, and incubated at 37 °C in a humidified atmosphere containing 5% CO_2_. Lipopolysaccharide (LPS, sourced from *E. coli* O111:B4) was used to stimulate the THP-1 cells. THP-1 cells (5 × 10^5^ cells/well) were pretreated with the soothing composition and Pt-liposome for 24 h, followed by LPS (1 μg/mL) for an additional 24 h. Total cellular RNA was extracted using the RaPure Total RNA Micro Kit according to the instructions. One microgram of total RNA was reverse-transcribed into cDNA using HiScript II Q RT SuperMix for qPCR (+gDNA wiper).

IL-8 mRNA expression was quantified by RT-qPCR, with GAPDH as the internal reference gene. Each reaction contained 2 μL of cDNA, PowerUp™ SYBR™ Green Master Mix, and 0.1 μM primers specific for IL-8 or GAPDH. Amplification was performed on an Applied Biosystems QuantStudio 1 system. Primer sequences were as follows:

IL-8:

Sense: 5’-ATACTCCAAACCTTTCCACCC-3’;

Antisense: 5’-AAACTTCTCCACAACCCTCTG-3’.

GAPDH:

Sense: 5’-GTCTCCTCTGACTTCAACAGCG-3’;

Antisense: 5’-ACCACCCTGTTGCTGTAGCCAA-3’.

All experiments were performed in triplicate (n = 3). Statistical analysis was conducted using one-way ANOVA followed by Tukey’s post hoc test, with *p* < 0.05 considered statistically significant.

**1.9 Efficacy test using SLS as irritant**

A skin irritation model was established on the forearms of four volunteers (n = 4) using a 24-h patch test with 200 μL of 0.5% sodium lauryl sulfate (SLS) as the irritant. Following patch removal, the control group received Solution B, while the test group received Solution A. Both formulations were applied twice daily to the irritated skin area.

Erythema Index (EI) and transepidermal water loss (TEWL) were measured at baseline (Day 0, D_0_) and on Days 1, 3, and 7 (D_1_, D_3_, D_7_) after treatment initiation. The soothing and reparative effects of the Pt-liposome combined with the soothing composition were evaluated by monitoring changes in EI and TEWL values. The rate of change (%) was calculated using the equation provided in the main text.

**1.10 Efficacy test after photorejuvenation**

This study was conducted as a single-center, single-blind, split-face randomized trial involving 30 healthy female participants aged 18-45 years (mean ± SD: 33.07 ± 6.47 years). All subjects underwent IPL photorejuvenation and were randomly assigned to receive the test facial mask on one side of the face and a basic moisturizer (control) on the other. Baseline assessments showed no statistical differences between sides in stratum corneum hydration, TEWL, or subjective symptoms (tightness, erythema, dryness, scaliness). Based on visual assessment and demographics, all participants were estimated to have Fitzpatrick skin phototypes III–IV, typical in East Asian populations. The study was approved by the institutional ethics board, registered in a national clinical trial registry, and conducted in accordance with the Declaration of Helsinki. Written informed consent was obtained from all participants.

Inclusion criteria required healthy female subjects aged 18-45 years with consistent redness scores on both sides of the face, dry or neutral skin, and no history of acne or melasma. Participants had to be willing to undergo photorejuvenation treatment, comprehend the test process, provide signed informed consent, and be available for the full duration of the study.

Exclusion criteria included: planned pregnancy, current pregnancy or lactation, or childbirth within the past 6 months; recent facial sun exposure (≤6 months) or prolonged outdoor exposure; photosensitive skin diseases (e.g., vitiligo, photosensitive dermatitis, lupus erythematosus) or unresolved inflammatory skin conditions (e.g., rosacea, seborrheic dermatitis, hormone-dependent dermatitis, contact dermatitis, atopic dermatitis); use of oral isotretinoin in the past 6 months; oily skin or acne tendency; melasma; photorejuvenation, laser, or other cosmetic treatments in the past 6 months; hypersensitivity to cosmetics, daily chemicals, protein products, alcohol, rubber, non-woven fabrics, or medications; serious systemic diseases, immune deficiencies, or autoimmune conditions; use of antihistamines in the past week or immunosuppressants in the past month; anti-inflammatory treatment at the test site in the past 2 months; participation in other clinical trials in the past 3 months; respiratory or dermatological treatments; and other medical conditions (e.g., coagulopathy, severe hyperglycemia, hypertension, hyperlipidemia, mental illness) that could affect test results. Individuals deemed unsuitable by clinical assessment were also excluded.

Throughout the testing period, participants were instructed to use only the prescribed test samples and to avoid any additional skincare products, medications, or cosmetic treatments, except for a permitted basic moisturizing lotion. They were advised to maintain a consistent, health-conscious daily routine and to minimize outdoor exposure during daylight hours. To ensure adherence to the split-face protocol, participants recorded and submitted videos of each application session. These recordings were reviewed by the study team to verify correct usage, and any deviations or instances of noncompliance were excluded from the final analysis. The testing environment was maintained at a controlled temperature of 21 ± 1 °C and a relative humidity of 50 ± 10%.

The left and right halves of the faces of the subjects were randomly assigned as the test side and the control side using a randomized table. The test side was treated with the facial mask for 15-20 min after photorejuvenation, followed by intermittent use of the facial mask within 14 days (three times a week). During the clinical test period, both the test and control sides were treated with a basic moisturizing lotion, which did not contain active ingredients, twice daily.

Subjects were screened based on predefined inclusion and exclusion criteria and provided signed informed consent prior to participation. On each visit to the laboratory, subjects were instructed to cleanse their face with a mild facial cleanser under the supervision of the researcher. After cleansing, they were to gently pat their face dry with a lint-free tissue. They were then seated in a climate-controlled room (21 ± 1°C, 50 ± 10% humidity) for 30 minwithout drinking water or consuming any beverages. During this time, they remained relaxed, avoided touching their face, and allowed their skin to acclimatize before any further assessments.

Prior to photorejuvenation, both subjective and objective assessments of skin condition were carried out. Subjective symptoms, including skin tightness, erythema (redness), dryness, and scaliness, were evaluated using a standardized 10-point questionnaire, with scores ranging from 0 (no symptoms) to 9 (severe symptoms). Both the researcher and the participant independently rated each parameter on the test and control sides of the face. Objective measurements included stratum corneum hydration, assessed with the CM825 device at the intersection of the outer eye corner and the lower nostril, and TEWL, measured using the Biox AquaFlux200 at the intersection of the mid-eye and nasal bridge. These quantitative metrics provided direct insight into skin barrier integrity.

Photorejuvenation was conducted by a qualified dermatologist using the Lumenis M22 intense light and laser skin treatment system. The system operated within a wavelength range of 515-1200 nm, with energy densities ranging from 10-18 J/cm^2^, which had been shown to be effective for improving skin texture and appearance. This standardized treatment protocol was applied to both the test and control sides of the faces of the participants.

Following photorejuvenation, participants underwent further subjective and objective assessments. Subjective evaluations were conducted using the same 10-point questionnaire to assess skin tightness, erythema, dryness, and scaliness at several time points: immediately after photorejuvenation (Day 0, AD_0_), 30 min post-application of the test sample on Day 1 (D_1T30min_), and on Day 3 (D_3_), Day 7 (D_7_), and Day 14 (D_14_) after product application. Objective assessments included re-measuring stratum corneum hydration using the CM825 and TEWL with the Biox AquaFlux200 at the same time points. These measurements provided a more detailed and quantitative understanding of how the test products influenced skin hydration and barrier integrity. The change in skin condition was expressed as percentage change from baseline using the formula provided in the main text.

Data analysis was performed using SPSS 25.0 statistical software. Measurement data were expressed as Mean ± SD (n = 30). Instrumental data were analyzed using paired t-tests or non-parametric tests depending on normality. Dermatological and self-evaluation scores were analyzed using the Wilcoxon non-parametric test. All hypothesis tests were conducted at a two-sided significance level of 0.05. A *p*-value of ≥0.05 indicated no statistically significant difference, while a *p*-value of <0.05 indicated a statistically significant difference. The following significance levels were used: ^*^*p* < 0.05, 0.001 ≤ ^**^*p* < 0.01, and ^***^*p* < 0.001. *p*-values ≥ 0.05 were denoted as “n.s” (not statistically significant) in the figures.





**Figure S1.** Size distribution of Pt particles measured by DLS





**Figure S2.** Size distribution of Pt-liposomes measured by DLS


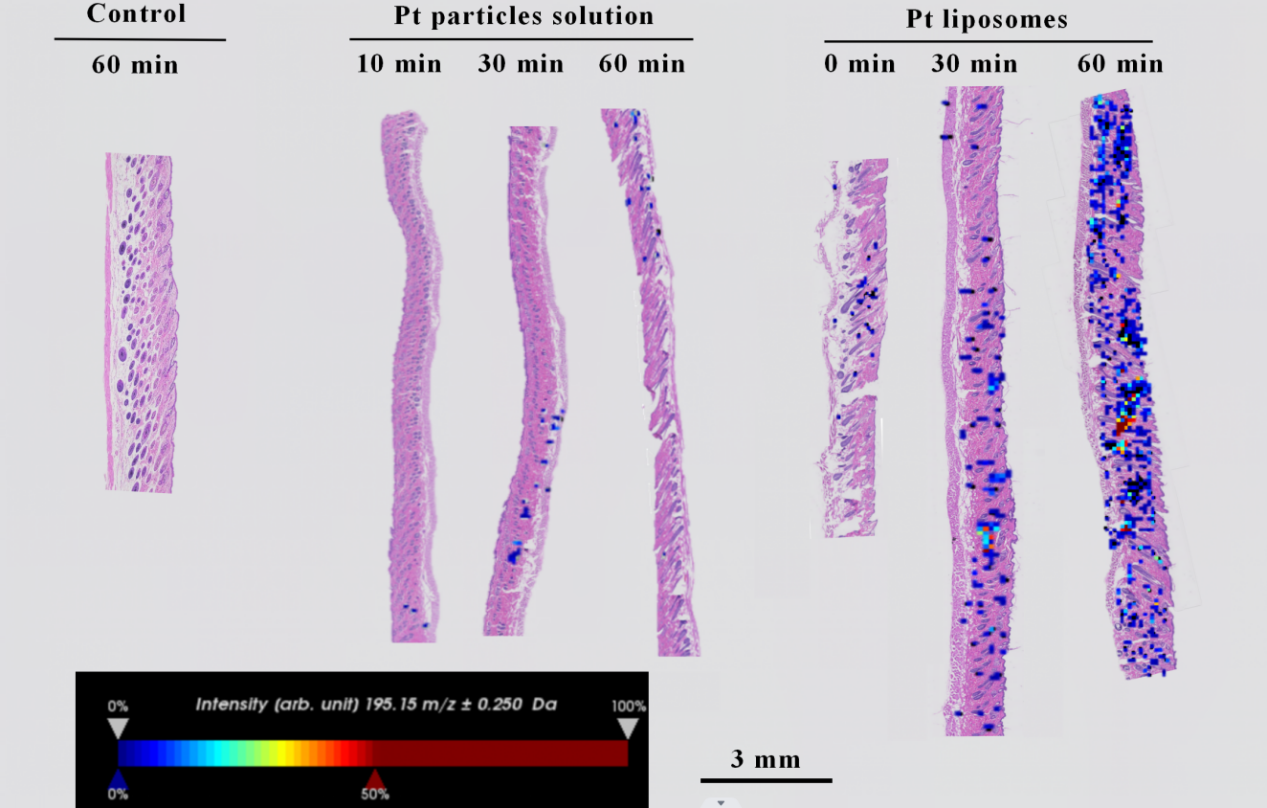


**Figure S3.** Mass spectrometry imaging (MSI) of mouse skin sections following topical application of Pt particles or Pt-liposomes for 10, 30, or 60 min, respectively. Pixel intensity maps depict the spatial distribution and relative abundance of platinum (Pt, 195 Da) within the tissue.


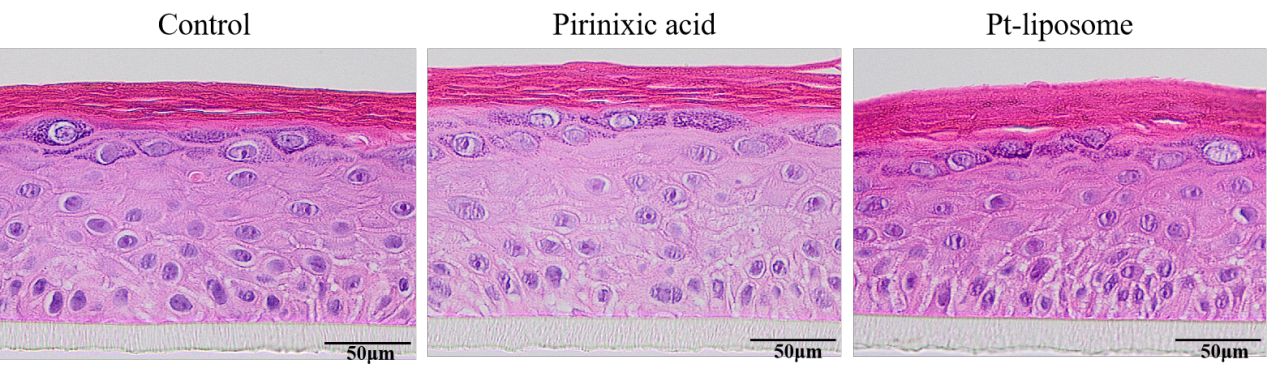


**Figure S4.** Histomorphometric analysis of 3D epidermal skin models after 24 h incubation with control, pirinixic acid, or Pt-liposome. Tissues were fixed in 4% paraformaldehyde for 24 h and stained with hematoxylin and eosin (H&E) for morphological evaluation.


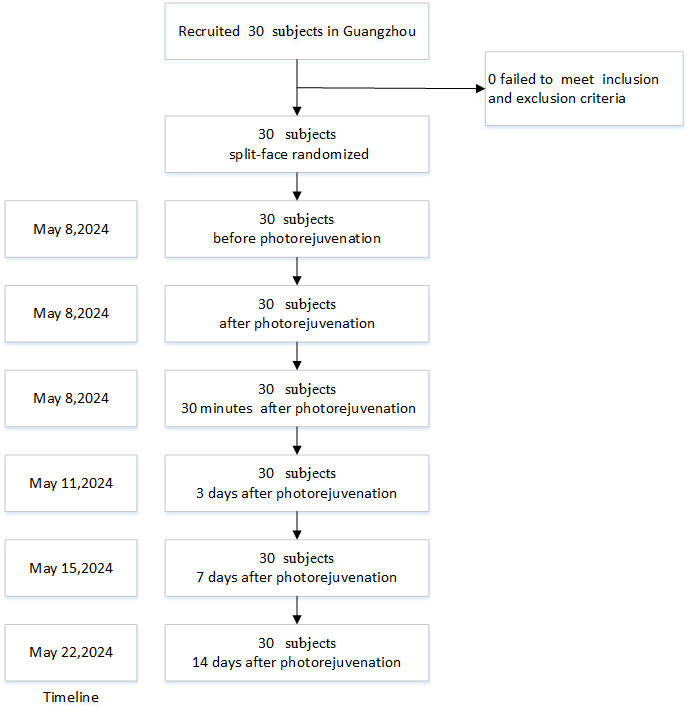


**Figure S5.** Flowchart of inclusion, randomization, and participant progression throughout the study.


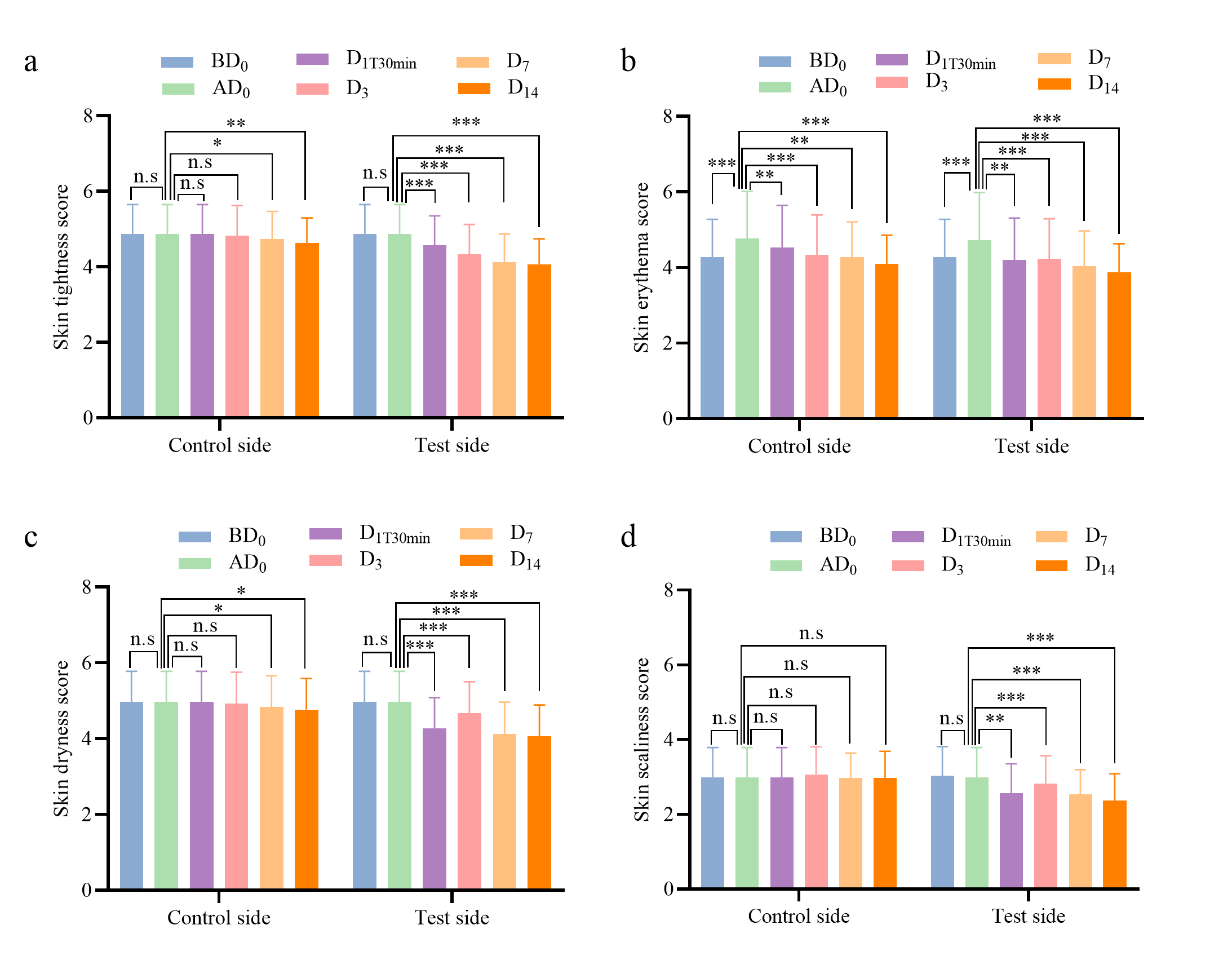


**Figure S6.** Subjective skin evaluation scores before and after photorejuvenation treatment. (a) Skin tightness score. (b) Skin erythema score. (c) Skin dryness score. (d) Skin scaliness score. Results are expressed as Mean ± SD. Statistical significance: ^*^*p* < 0.05, ^**^*p* < 0.01, ^***^*p* < 0.001; n.s., not statistically significant.

**Table S1.** Relative distribution of platinum (Pt) in skin tissue at different time points following topical application.

| Groups | Time (min) | Relative Pt distribution |
| --- | --- | --- |
| Control | 60 | 0 |
| Pt particles | 10 | 0.1 |
|  | 30 | 1.3 |
|  | 60 | 3.3 |
| Pt-liposome | 10 | 2.5 |
|  | 30 | 9.5 |
|  | 60 | 50 |

Note: Relative distribution values were calculated based on mass spectrometry imaging signal intensities (arbitrary units). Higher values indicate greater platinum penetration into the skin. The control group used propylene glycol only.

**Table S2.** Changes in erythema index (EI) values at different time points in the control and test groups (Mean ± SD).

| Time | Control group | | Test group | |
| --- | --- | --- | --- | --- |
|  | EI value | Rate of change (%) | EI value | Rate of change (%) |
| D_0_ | 259.00±40.14 | - | 256.33±45.77 | - |
| D_1_ | 261.25±22.98 | 0.87 | 249.33±56.20 | -2.73 |
| D_3_ | 247.33±31.84 | -4.51 | 222.25±26.09 | -13.30 |
| D_7_ | 231.42±35.11 | -10.65 | 212.75±21.19 | -17.00 |

Note: The rate of change (%) is calculated relative to Day 0 (D_0_) for each group.

**Table S3.** Changes in transepidermal water loss (TEWL) values at different time points in the control and test groups (Mean ± SD).

| Time | Control group | | Test group | |
| --- | --- | --- | --- | --- |
|  | TEWL (g/(h·m^2^)) | Rate of change (%) | TEWL (g/(h·m^2^)) | Rate of change (%) |
| D_0_ | 19.40±4.16 | - | 22.65±6.53 | - |
| D_1_ | 21.55±5.44 | 11.08 | 19.28±5.79 | -14.88 |
| D_3_ | 15.60±1.28 | -19.59 | 13.28±4.12 | -41.37 |
| D_7_ | 11.53±0.54 | -40.57 | 10.00±2.57 | -55.85 |

Note: The rate of change (%) is calculated relative to Day 0 (D_0_) for each group.
